# The hTERT-p50 homodimer inhibits PLEKHA7 expression to promote gastric cancer invasion and metastasis

**DOI:** 10.1038/s41388-023-02630-9

**Published:** 2023-02-23

**Authors:** Yu-Yun Wu, Yu-Feng Xiao, Li-Xing Tian, Bing He, Jiao Liu, Zhi-Bin Li, Huan Yang, Yang Chen, Qiang Luo, Bo-Sheng Li, Shi-Ming Yang

**Affiliations:** 1 Department of Gastroenterology, Xinqiao Hospital, Army Medical University (Third Military Medical University), Chongqing, 400037 China; 2 Department of Endoscope, The General Hospital of Shenyang Military Region, Shenyang, 110016 China; 3 Chongqing Municipality Clinical Research Center for Gastroenterology, Chongqing, 400037 China

**Keywords:** Metastasis, Gastric cancer

## Abstract

Although accumulating evidence has highlighted the molecular mechanisms by which hTERT promotes tumour cell invasion and metastasis, the molecular mechanisms of the properties enabling hTERT to contribute to invasion and metastasis have not been clearly illustrated. Here, we report that hTERT promotes gastric cancer invasion and metastasis by recruiting p50 to synergistically inhibit PLEKHA7 expression. We observed that the expression of PLEKHA7 in gastric cancer was significantly negatively associated with the TNM stage and lymphatic metastasis and that decreased PLEKHA7 expression dramatically increased invasion and metastasis in gastric cancer cells. Further mechanistic research showed that hTERT directly regulates PLEKHA7 expression by binding p50 and recruiting the hTERT/p50 complex to the PLEKHA7 promoter. Increased hTERT dramatically decreased PLEKHA7 expression and promoted invasion and metastasis in gastric cancer cells. The hTERT-mediated invasion/metastasis properties at least partially depended on PLEKHA7. Our work uncovers a novel molecular mechanism underlying invasion/metastasis in gastric cancer orchestrated by hTERT and p50.

## Introduction

Through its very specific and requisite TTAGGG repeat, human telomerase reverse transcriptase (hTERT), a core component of telomerase holoenzymes, contributes to telomere DNA synthesis to maintain the telomere length [1–4]. hTERT is absent in most human somatic cells but is expressed in stem cells [5, 6]. Importantly, hTERT expression is reactivated in 80–90% of all cancers [7]. Moreover, in cancer cells, hTERT expression not only exhibits canonical functions in telomere stabilization but also regulates many processes involved in increased cell proliferation, antiapoptotic effects and increased migration/invasion [8, 9]. Accumulating evidence has highlighted the telomere-independent functions of hTERT, including its roles in mitochondrial functions [10, 11], NF-κB-dependent transcription [12], the DNA damage response [13–15], and Foxo3a ubiquitination degradation [16]. Increasing evidence has also suggested that hTERT is highly expressed in many human cancers, including gastric cancer (GC), and is positively correlated with tumour aggressiveness [17–19]. However, the molecular mechanisms linking the telomere-independent function of hTERT to gastric cell invasion/metastasis phenotypes remain unclear.

The epithelial cell junction complex is composed of tight junctions (TJs), adherens junctions (AJs) and desmosomes on the apical side of the cell-cell junction [20, 21]. AJs are organized by cadherins and nectin and associated cytoplasmic proteins, including α-catenin, β-catenin and p120-catenin. These cadherins associate with catenin protein family members and are critical mediators of AJ function and stability [20]. AJs regulate cell adhesion and morphogenesis by forming “zonula adherens” (ZA) or “adhesive band” complexes [22–24]. The loss of AJ integrity results in developmental abnormalities and pathological conditions [25, 26]. Recent studies have identified a novel protein, pleckstrin homology domain-containing family A member 7 (PLEKHA7) [27], that links the microtubule cytoskeleton to ZA junctions by binding p120 and nezha [22, 28, 29]. The depletion of PLEKHA7 expression in colon cancer cells has been shown to disrupt ZA organization [24], suggesting that PLEKHA7 may maintain AJ integrity in epithelial cells. PLEKHA7 plays a remarkable functional role in suppressing SNAI1, MYC, and CCND1 expression by regulating the biogenesis of the microRNAs (miRNAs) miR-24, let-7 g, miR-30a and miR-30b, ultimately inhibiting anchorage-independent growth by recruiting the microprocessor complex to ZA junctions [30]. Therefore, PLEKHA7 is involved in not only stabilizing adhesive protein complexes but also important physiological and pathological processes. The loss of PLEKHA7 expression disrupts the interaction of the microprocessor complex at ZA junctions, resulting in a decreased expression of tumour-suppressive miRNAs and an increased expression of certain miRNAs and genes related to carcinogenesis, tumour growth and/or migration [30–35]. Furthermore, PLEKHA7 is gradually lost in certain tumours during tumour progression, suggesting that PLEKHA7 deficiency is a common mechanism of tumour development in these tumours. However, the molecular mechanisms regulating PLEKHA7 in tumour progression to malignancy are unclear.

In this report, we illustrate the mechanism by which PLEKHA7 is gradually lost during GC progression. Specifically, in vitro and in vivo experimental results showed that PLEKHA7 was gradually lost during tumour progression to malignancy in GC and that PLEKHA7 deficiency exacerbated tumour invasion and metastasis. Moreover, this regular loss of PLEKHA7 expression was accompanied by an increase in hTERT/p50 complexes bound to the PLEKHA7 promoter. Furthermore, we show that hTERT enhanced hTERT/p50 complex binding to the PLEKHA7 promoter, which inhibited PLEKHA7 expression.

## Results

### PLEKHA7 deficiency is an indicator of a poor prognosis in stomach adenocarcinoma patients

**Fig. 1:**
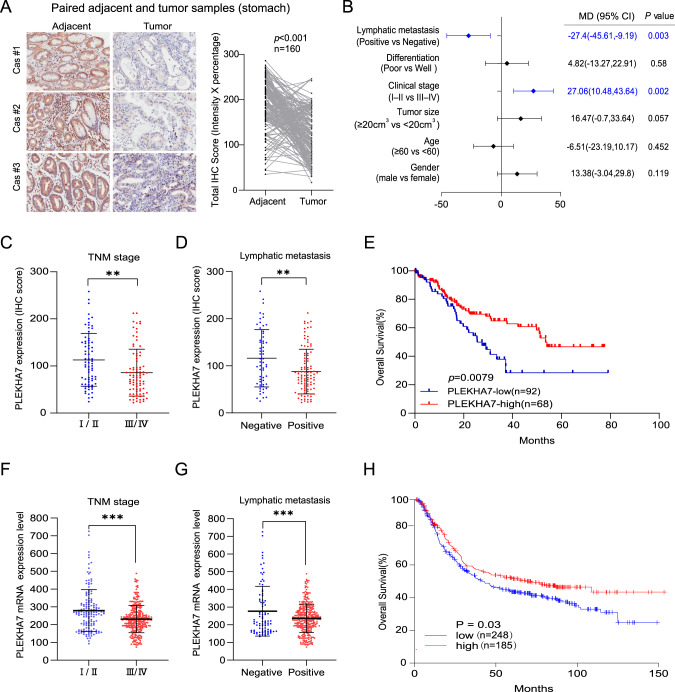
Low PLEKHA7 expression is a negative prognostic factor indicating a higher pathological stage and more frequent distant metastasis in stomach adenocarcinoma patients. **A** Typical PLEKHA7 staining in 160 paired adjacent and stomach adenocarcinoma tissue samples (left) and statistical results of the staining scores (right). The scores were calculated as the staining intensity score×the percentage of stained cells (*p* < 0.001). **B** Multivariate analysis showing the correlation between clinical characteristics and PLEKHA7 expression (*n* = 160). Significant elements are highlighted in blue. **C**, **D** The association between PLEKHA7 expression and the TNM stage/lymphatic metastasis in gastric cancer (GC) patients (*n* = 160). **E** Survival analysis showing that overall, patients with low PLEKHA7 expression exhibited lower overall survival than those with high PLEKHA7 expression. **F**, **G** The association between PLEKHA7 expression and the TNM stage/lymphatic metastasis in GC patients in the GSE84437 dataset (*n* = 433). **H** Survival analysis of the GSE84437 dataset showing that overall, patients with low PLEKHA7 expression displayed lower overall survival than those with high PLEKHA7 expression.

To identify tumour tissues that show the most significant differences in PLEKHA7 expression, we performed immunohistochemistry (IHC) staining to detect the PLEKHA7 expression levels in tissue samples of common types of cancers (lung adenocarcinoma (LUAD), colon adenocarcinoma (COAD), liver hepatocellular carcinoma (LIHC), kidney renal clear cell carcinoma (KIRC), prostate adenocarcinoma (PRAD), stomach adenocarcinoma (STAD), and breast cancer (BC)) and compared these levels to those in paired normal tissues. Compared with the respective paired normal tissues, PLEKHA7 expression was the most significantly different in the gastric tumour tissues (Fig. S1A). To confirm this finding, PLEKHA7 expression was detected in gastric tumours and paired adjacent tissues (*N* = 160) by IHC staining. We found that the PLEKHA7 level in the GC tissues was significantly lower than that in the Adjacent tissues (*P* < 0.001) (Fig. 1A).

After identifying the most significant difference in PLEKHA7 expression between gastric tumour tissues and adjacent tissues, we analysed the effect of PLEKHA7 on the prognosis of GC patients. All 160 GC patient tissues were stratified into subgroups according to PLEKHA7 expression. Moderate and highly intense PLEKHA7 IHC staining was considered indicative of a high PLEKHA7 expression level, and no or poor intensity PLEKHA7 IHC staining was considered indicative of a low PLEKHA7 expression level (Fig. S1B). We further analysed the correlation between the clinical characteristics and PLEKHA7 expression in GC patients. A multivariate analysis was performed to evaluate the clinical pathology data, and the results revealed that PLEKHA7 expression was significantly correlated with lymphatic metastasis (mean difference [MD] = −27.4, 95% confidence interval [CI] = −45.61–−9.19, *P* = 0.003) and the clinical stage (MD = 27.06, 95% CI = 10.48–43.64, *P* = 0.002) (Fig. 1B). To verify the analysis results, the patients were stratified into subgroups according to their pathological stage and whether lymphatic metastasis was evident. The expression of PLEKHA7 significantly differed between early-stage (stages I and II) and late-stage (stages III and IV) disease (Fig. 1C, F), and a gradual decrease in PLEKHA expression was found to be associated with more severe pathological grades (Fig. S1C). As expected, PLEKHA7 expression remained significantly reduced in the lymphatic metastasis group compared with that in the nonlymphatic metastasis group (Figs. 1D, G and S1D). The survival analysis revealed that low PLEKHA7 expression was specifically associated with low overall survival (*P* < 0.001) (Fig. 1E, H). In summary, our data suggest that PLEKHA7 expression is negatively associated with GC progression, indicating that PLEKHA7 deficiency may be critical for a poor prognosis in GC patients.

### Silencing PLEKHA7 increases GC cell invasion/migration and promotes tumour metastasis in vitro and in vivo

**Fig. 2:**
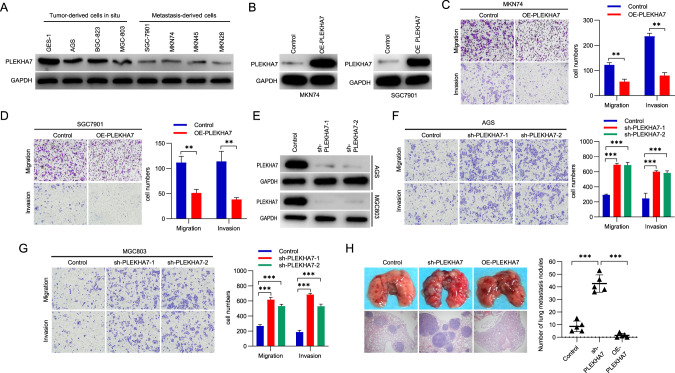
PLEKHA7 downregulation enhances gastric cancer (GC) cell invasion and migration. **A** PLEKHA7 expression levels in different GC cell lines and immortalized gastric epithelial cells (GES-1). **B** PLEKHA7 overexpression (OE-PLEKHA7) in MKN74 and SGC7901 GC cell lines. **C**, **D** Transwell assays were applied to detect the migration and invasion abilities of MKN74 and SGC7901 cells after PLEKHA7 overexpression. **E** PLEKHA7 knockdown in MKN74 and SGC7901 GC cell lines. **F**, **G** Transwell assays were applied to detect the migration and invasion abilities of AGS and MGC803 cells after PLEKHA7 knockdown. **H** A nude mouse metastasis model was established by intravenous injection of PLEKHA7-overexpressing MKN74 cells or PLEKHA7-knockdown MKN74 cells and control vector-expressing MKN74 cells. A significantly increased number of metastatic nodules was observed in the lungs in the PLEKHA7-knockdown group compared with that in the control group, but the number was lower in the PLEKHA7-overexpression group.

To determine the importance of PLEKHA7 expression in GC cell invasion/metastasis, we measured the PLEKHA7 protein levels in GC cells in situ and those derived from metastatic liver or lymphatic tissue and found lower PLEKHA7 expression levels in the metastasis-derived cells than in the cell lines in situ (Fig. 2A). Therefore, for the PLEKHA7 overexpression (OE-PLEKHA7) experiment, metastasis-derived GC cell lines (MKN74 and SGC7901) were infected with lentivirus expressing PLEKHA7 to assess the effect of PLEKHA7 on GC cell migration and invasion ability (Fig. 2B). Compared with the negative control group, PLEKHA7 overexpression significantly reduced the invasion/migration ability of MKN74 and SGC7901 cells (Fig. 2C and D). To further confirm whether PLEKHA7 plays a critical role in negatively regulating migration/invasion ability of gastric cancer cell, we applied genetic methods to deplete endogenous PLEKHA7 in cells. Depletion of endogenous PLEKHA7 in two GC cell lines (AGS and MGC803) with high endogenous PLEKHA7 expression using two independent shRNAs or sgRNAs (Fig. 2E and Fig. S2A, B), resulting in the invasion/migration ability of these two cell lines was significantly increased compared with that in the control group after depleting PLEKHA7 (Fig. 2F, G and Fig. S2C).

We also performed a mouse metastasis model experiment to investigate the metastatic effect of PLEKHA7 expression in vivo. MKN74 cells with OE-PLEKHA7 or PLEKHA7 knockdown (sh-PLEKHA7) were injected into the tail vein of nude mice. The number of metastatic lung nodules in the knockdown group was significantly increased (*P* < 0.001) compared with that in the NC group. In contrast, a few metastatic nodules were found in the lungs of the mice in the OE-PLEKHA7 group (Fig. 2H). These results indicate that PLEKHA7 deficiency is associated with metastasis in GC.

### p50 suppresses PLEKHA7 transcriptional expression

To understand the regulatory mechanism of PELKHA7 expression, we treated gastric cancer cells (MKN74) and immortalized gastric epithelial cells (GES-1) with cycloheximide D and MG-132, respectively, and detected PLEKHA7 expression at different times. The results showed no difference in either the degradation or stabilization of the PLEKHA7 protein level in the two cell lines (Fig. S3A–D). We also evaluated PLEKHA7 mRNA stability in MKN74 and GES-1 cells. The results showed no difference in PLEKHA7 mRNA stability between MKN74 and GES-1 cells (Fig. S3E). Furthermore, our nuclear run-on assays suggested that the difference in PLEKHA7 expression between GC cell (MKN74) and immortalized gastric epithelial cell (GES-1) is likely due to transcriptional inhibition (Fig. S3F). These results confirm that the regulation of PLEKHA7 in GC is not caused by mRNA or protein stabilization and is presumably regulated at the transcriptional level.

**Fig. 3:**
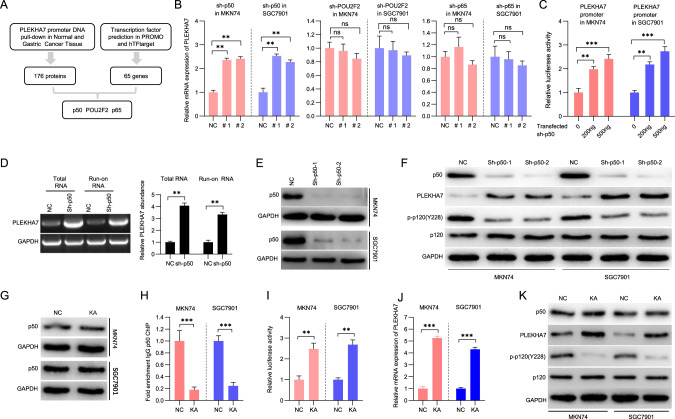
p50 was identified as a transcriptional repressor of PLEKHA7 in gastric cancer (GC) cells. **A** Schematic showing an integration of the analyses of the data from two aspects, including PLEKHA7 promoter DNA pull-down proteins and the prediction of the transcription factors binding the PLEKHA7 promoter. **B** PLEKHA7 mRNA abundance was detected by RT‒qPCR after the knockdown of p50, POU2F2 or p65. **C** Luciferase reporter assay. Knocking down p50 expression (sh-p50) enhanced the luciferase activity of the PLEKHA7 promoter compared with nontransfected shRNA. **D** Determination of the PLEKHA7 transcription initiation rate via a nuclear run-on assay in MKN74 cells after p50 was transfected with shRNA for 72 h. **E** Knockdown of p50 in MKN74 and SGC7901 GC cell lines. **F** The expression of PLEKHA7 and its downstream genes (p120 and p-p120(Y228)) was detected by a western blot analysis after p50 knockdown. **G** p50 expression in cells treated with PBS or 50 µM kamebakaurin (KA) was detected by a western blot analysis. **H** Chromatin immunoprecipitation (ChIP) analysis of p50 recruitment to the PLEKHA7 promoter in MKN74 and SGC7901 cells treated with PBS (NC) or 50 µM KA. **I** Luciferase reporter assay. PLEKHA7 promoter transcriptional activity increased after treatment with 50 µM KA compared with that after treatment with PBS (NC). **J** PLEKHA7 mRNA abundance was detected by RT‒qPCR in MKN74 and SGC7901 cells treated with PBS (NC) or 50 µM KA. **K** The expression of PLEKHA7 and its downstream genes (p120 and p-p120(Y228)) in cells treated with PBS (NC) or 50 µM KA was detected by a western blot analysis.

Previous studies have demonstrated that gene transcription is regulated by various transcription factors [8]. To identify the factors that regulate PLEKHA7 expression in GC, we focused on the proteins that bind the PLEKHA7 promoter. To identify these transcription factors, we applied the following two methods: (1) biotin labelling of the “core promoter” DNA bait in PLEKHA7 (located within a region -2000 and +200 bp from the PLEKHA7 transcription start) for use in DNA pull-down assays (Fig. S4A and B), followed by a mass spectrometry analysis, and through this method, 176 proteins were found, and (2) sixty-five transcription factors were identified from a screening of two transcription factor prediction websites (hTFtarget and TRANSFAC). Next, we identified 3 candidate genes, p50, POU2F2 and p65, based on the crossover of the two gene clusters initially identified (Fig. 3A). Among these potential regulators, only p50 was pulled down in MKN74 cells in the DNA pull-down assay (Fig. S4D). We also observed that PLEKHA7 expression increased only after p50 expression was knocked down (Fig. 3B and Fig. S5). These results suggest that p50 may be a key regulator of PLEKHA7, but not POU2F2 or p65. Then, we sought to determine whether p50 is associated with PLEKHA7 transcriptional regulation. Our dual-luciferase reporter assays suggested that PLEKHA7 promoter luciferase activity gradually increased with increasing transfection of p50 shRNA compared with that in the nontransfected group (Fig. 3C). To confirm this result, the effect of p50 knockdown on PLEKHA7 transcription was assessed. We found that the p50-mediated regulation of PLEKHA7 expression is likely due to transcriptional inhibition (Fig. 3D). The western blot analysis showed a remarkable increase in PLEKHA7 in the p50 knockdown cells compared with that in the p50 nonknockdown cells (Fig. 3E, F). In addition, the expression of phosphorylated p-p120 (Y228), which is downstream of PLKEHA7, was significantly reduced [33] (Fig. 3F). Similar to the effects mediated by knocking down p50, the PLEKHA7 promoter luciferase activity, mRNA levels and protein levels were enhanced by treatment with kamebakaurin (KA) compared with those following treatment with DMSO (Fig. 3I–K), which inhibits p50 binding to DNA (Fig. 3G, H). Taken together, these data demonstrate that p50 is a major suppressor of PLEKHA7 in GC cells. Interestingly, the forced expression of p50 did not substantially decrease the PLEKHA7 mRNA or protein levels in MKN74 cells (Fig. S5A, B). Similar to the lack of change in the mRNA and protein levels, the transcriptional activity of the PLEKHA7 promoter was also not obviously changed (Fig. S5C, D). These results indicate that other mechanisms cooperate with p50 to regulate PLEKHA7 expression.

### hTERT necessarily binds p50 to inhibit PLEKHA7 expression

**Fig. 4:**
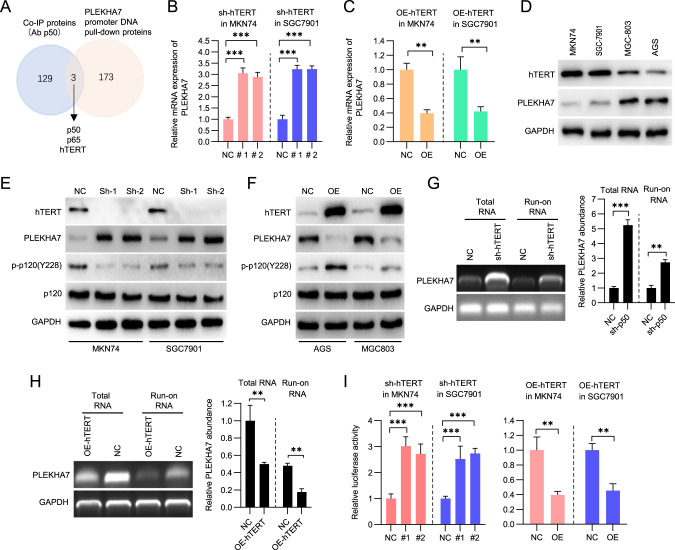
hTERT inhibits PLEKHA7 expression in gastric cancer (GC) cells. **A** Schematic showing an integration of the analyses of the data from two aspects, including the Co-IP and PLEKHA7 promoter DNA pull-down proteins. **B**, **C** PLEKHA7 mRNA abundance was detected by RT‒qPCR after the knockdown and overexpression of hTERT. **D** hTERT and PLEKHA7 protein expression levels in MKN74, SGC7901, MGC803 and AGS. The expression of PLEKHA7 and its downstream genes (p120 and p-p120(Y228)) was detected by a western blot analysis after hTERT knockdown (**E**) and hTERT overexpression (**F**). Determination of the PLEKHA7 transcription initiation rate via a nuclear run-on assay in MKN74 cells after hTERT knockdown (**G**) and hTERT overexpression (**H**) for 72 h. **I** Luciferase reporter assays were used to analyse the PLEKHA7 promoter transcriptional activity after hTERT knockdown (left) and hTERT overexpression (right) in MKN74 and SGC7901 cells.

To identify p50 cooperators that regulate PLEKHA7 expression, we first performed coimmunoprecipitation (Co-IP) to pull down potential proteins that bind p50 in normal and GC tissues. The mass spectrometry analysis revealed that 132 proteins putatively interact with p50 (Fig. 4A). Then, we identified 176 proteins that were pulled down by the PLEKHA7 promoter. The intersection analysis showed that only p50, p65 and hTERT existed in both sets of proteins (Fig. 4A). The RT‒qPCR and western blot experiments demonstrated that the PLEKHA7 mRNA and protein levels were increased in the hTERT-knockdown, but not the p65-knockdown, tumour cells compared with those in the control-knockdown tumour cells (Figs. 4B, E, 3B and S5G). In contrast, the PLEKHA7 mRNA and protein levels were decreased in the hTERT-overexpressing, but not the p65-overexpressing, tumour cells compared with those in the control-overexpressing tumour cells (Figs. 4C, F and S5E). Importantly, the nuclear run-on assays showed that the hTERT-regulated expression of PLKEHA7 can likely be attributed to PLEKHA7 transcriptional initiation (Fig. 4G, H). The validation by the dual-luciferase reporter assays showed that the hTERT-knockdown cells exhibited heightened luciferase activity compared with the control-knockdown cells (Fig. 4I). Altogether, our data suggest that hTERT is an upstream regulator of PLEKHA7 expression.

**Fig. 5:**
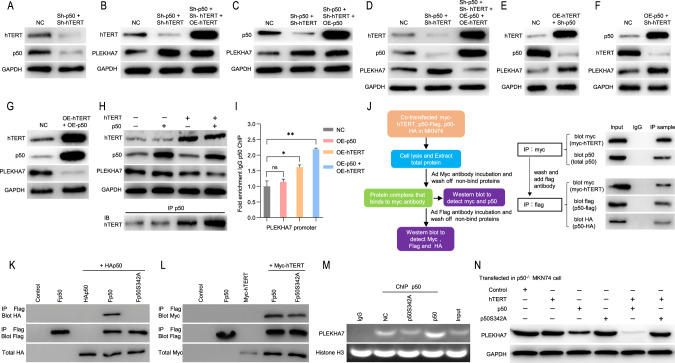
hTERT complexed with p50 homodimers regulates PLEKHA7. **A** hTERT and p50 expression was analysed by a western blot analysis after the knockdown of both. **B** hTERT and PLEKHA7 expression was analysed by a western blot analysis after the knockdown of p50 and hTERT and then rescued hTERT expression. **C** p50 and PLEKHA7 expression was analysed by a western blot analysis after the knockdown of p50 and hTERT and then rescued p50 expression. **D** hTERT, p50 and PLEKHA7 expression was analysed by a western blot analysis after the knockdown of p50 and hTERT and then rescued both hTERT and p50 expression. **E** hTERT, p50 and PLEKHA7 expression was analysed by a western blot analysis after the knockdown of p50 and overexpression of hTERT. **F** p50, hTERT and PLEKHA7 expression was analysed by a western blot analysis after the knockdown of hTERT and overexpression of p50. **G** hTERT, p50 and PLEKHA7 expression was analysed by a western blot analysis after the overexpression of both hTERT and p50. **H** hTERT, p50 and PLEKHA7 expression was analysed by a western blot assay and hTERT association with p50 was analysed by a co-IP assay with an anti-p50 antibody after various treatments. **I** Chromatin immunoprecipitation (ChIP) assay of p50 recruitment to the PLEKHA7 promoter in MKN74 cells after various treatments. **J** Schematic showing the two-step Co-IP assay (left), and hTERT association with the p50 homodimer was analysed by Co-IP (right). **K** Representative western blots showing that p50 does not bind p50S342A. **L** western blots showing that p50S342A retains its interaction with hTERT. **M** ChIP-PCR assay of p50 and p50S342A recruitment to the PLEKHA7 promoter. **N** western blots showing that hTERT complexed with p50 homodimers to regulate PLEKHA7 expression.

Previous research proposed a model in which hTERT acts as a cofactor to regulate gene expression [2]. We speculate that PLEKHA7 expression is regulated by hTERT in a manner similar to that previously reported. After the p50 or hTERT overexpression in the p50 and hTERT knockdown cells, the PLEKHA7 protein levels remained restored compared with those in the p50 and hTERT knockdown cells (Fig. 5A–C). However, when both p50 and hTERT were simultaneously overexpressed in the p50 and hTERT knockdown cells, the PLEKHA7 protein levels were greatly diminished compared with those in the p50 and hTERT knockdown cells (Fig. 5D). We also observed that the trends in the PLEKHA7 protein levels were consistent (an increase) regardless of whether hTERT was overexpressed in p50-knockdown cells or p50 was overexpressed in hTERT-knockdown cells compared with those in the control-knockdown cells (NC) (Fig. 5E, F). Moreover, PLEKHA7 expression decreased only after simultaneous hTERT and p50 overexpression (Fig. 5G). These results indicate that hTERT and p50 may act as a complex to regulate PLEKHA7. Subsequently, both p50 and hTERT were overexpressed in MKN74 cells and the cells were collected for Co-IP and ChIP experiments. As hTERT and p50 binding increased, the p50 bound to the PLEKHA7 promoter gradually increased, and the expression of PLEKHA7 gradually decreased (Fig. 5H, I). These results suggest that hTERT and p50 form a complex to suppress PLEKHA7 expression.

### hTERT complexed with p50 homodimers regulates PLEKHA7 expression

Previous research reported that the suppressive effects of p50 on gene expression are mediated by p50 homodimers [36]. We speculate that the hTERT/p50 complex inhibits PLEKHA7 expression through p50 homodimer-mediated inhibition. In a two-step IP experiment with cells cotransfected with Myc-hTERT, Flag-p50 and HA-p50 (Fig. S6A–C), IP was initially performed with Myc-hTERT to isolate the complex, followed by washing and the addition of an anti-Flag antibody to isolate the remaining complexes. Finally, a western blot assay was performed to detect Myc-hTERT, Flag-p50 and HA-p50. The western blot analysis of the final IP complexes confirmed that the hTERT and p50 dimers form a complex (Fig. 5J). To obtain further evidence showing that the hTERT/p50:p50 complex regulates PLEKHA7 expression, we introduced a mutant in which an alanine was introduced to replace the evolutionarily conserved residue Ser342 in Flag-p50 (Fp50S342A) (Fig. S6D, E) and failed to form the p50 homodimer for use in the subsequent experiments according to a previous report [36]. The Co-IP assays demonstrated that the mutant p50 (Fp50S342A) did not interact with wild-type p50 (HA-p50) (Fig. 5K), but did not affect its interaction with hTERT (Fig. 5L). However, the ChIP-PCR experiments showed that mutant p50 (Fp50S342A) disrupted endogenous p50 binding to the PLEKHA7 promoter (Fig. 5M). This result indicates that Fp50S342A interfered with p50:p50 dimer formation, preventing the hTERT:p50:p50 complex from binding the PLEKHA7 promoter. As expected, wild-type p50 cotransfected with hTERT in p50^−/−^ MKN74 cells significantly suppressed PLEKHA7 expression, but this effect was not observed in the Fp50S342A+hTERT group (Fig. 5N and Fig. S6F, G). These results confirm that hTERT complexed with p50 homodimers (hTERT:p50:p50) to suppress PLEKHA7 expression.

### hTERT and p50 directly combine with the PLEKHA7 promoter in GC cells to synergistically suppress PLEKHA7 expression

**Fig. 6:**
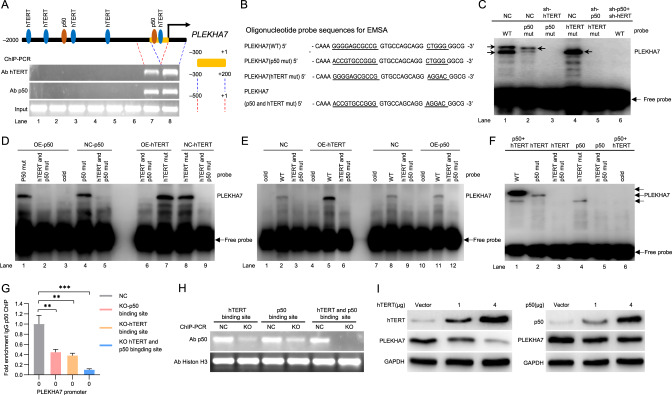
hTERT and p50 directly bind the PLEKHA7 promoter in GC cells and synergistically inhibit PLEKHA7 expression. **A** Schematic showing the position of p50 and hTERT binding to the PLEKHA7 promoter. **B** Sequences of oligonucleotide probes used in the EMSAs. **C** EMSAs were performed using nuclear extracts from MKN74 cells with the knockdown of hTERT or knockdown of p50 on the PLEKHA7 promoter. **D**, **E** EMSAs were performed using nuclear extracts from MKN74 cells with forced hTERT or p50 expression on the PLEKHA7 promoter. **F** EMSA supershift analyses were performed with the indicated antibodies on the PLEKHA7 promoter. **G**, **H** Chromatin immunoprecipitation (ChIP) and ChIP-PCR analysis showing p50 binding to the PLEKHA7 promoter in MKN74 cells in which the binding sequences of p50, hTERT or both p50 and hTERT were knocked out. **I** PLEKHA7 expression after cells were transfected with different amounts of hTERT or p50.

To clarify the effect of each component of the hTERT:p50:p50 complex on PLEKHA7 expression, we predicted two p50-binding regions (from −261 to −251 and from −1238 to −1228) on the PLEKHA7 promoter through a bioinformatics analysis (Fig. 6A). Subsequently, we used eight pairs of primers to amplify different regions of the PLEKHA7 “core promoter” in the samples obtained from the ChIP experiments. Regardless of whether the sample was pulled down by targeting p50 or hTERT, only two regions (from −500 to +1 and from −300 to +200) containing one of the speculated p50-binding regions (from −261 to −251) were amplified (Fig. 6A and S7A). In addition, we analysed the intersection sequence and found that the “CTGGG” sequence near the putative p50-binding sequence (“GGGGAGCGCC”) was extremely similar to the hTERT-binding sequence (“TTGGG”) [12]. Therefore, we hypothesized that the “CTGGG” sequence may be an hTERT-binding sequence in the PELKHA7 promoter. Next, a luciferase reporter assay was performed to evaluate the contribution of the “GGGGAGCGCC” sequence (the putative p50-binding sequence) and the “CTGGG” sequence to p50 and hTERT binding to the PLEKHA7 promoter. We observed that regardless of whether the “GGGGAGCGCC” sequence was replaced with the “GGGACTTTCC” sequence (the consensus p50-binding sequence) or whether the “CTGGG” sequence was replaced with the “TTGGG” sequence (hTERT-binding sequence) [12] in the promoter (Fig. S7C–H), the luciferase activity did not differ from that with the WT sequence (Fig. S7B). However, the activity of luciferase was significantly increased regardless of whether the “GGGGAGCGCC” sequence or the “CTGGG” sequence was mutated, and this effect was the most pronounced when both sequences were mutated compared with the WT sequence (Fig. S7B). These data indicate that “GGGGAGCGCC” and “CTGGG” are the key sequences where p50 and hTERT bind the PLEKHA7 promoter.

Then, an electrophoretic mobility shift assay (EMSA) was used to analyse the interaction between hTERT/p50 and the PLEKHA7 promoter. When the PLEKHA7 promoter oligonucleotide containing the p50 and hTERT binding sites acts as a probe (WT), two notable mobility shifts were visualized in control-knockdown cell lysates (Fig. 6C, lane 1); however, these shifts were not evident in the p50 and hTERT knockdown cell lysates (Fig. 6C, lane 6). When the PLEKHA7 promoter oligonucleotide containing only p50 or hTERT binding sites acted as a probe (p50 mut or hTERT mut), there was only one mobility shift in the control-knockdown cell lysates (Fig. 6C, lanes 2 and 4) and no mobility shift in the p50 or hTERT knockdown cell lysates (Fig. 6C; lanes 3 and 5). To investigate the contribution of the “GGGGAGCGCC” sequence to p50 binding, we used the PLEKHA7 promoter oligonucleotide in which the “GGGGAGCGCC” sequence was mutated (p50 mut) but retained the hTERT putative binding site (“CTGGG” sequence). Indeed, the PLEKHA7 promoter with a mutant p50 binding site showed no difference in the level of p50 binding in the p50-overexpressing cell lysates compared with that in the control-overexpression cell lysates (Fig. 6D, comparing lanes 1 and 4). Similar changes were observed in the PLEKHA7 promoter with a mutant hTERT binding site in a set of hTERT overexpression versus control-overexpression cell lysates (Fig. 6D, comparing lanes 7 and 8). These results indicate that the “GGGGAGCGCC” and “CTGGG” sequence on the PLEKHA7 promoter are the binding sites of p50 and hTERT, respectively. Importantly, the PLEKHA7 promoter showed a highlighted level of binding in the hTERT-overexpressing cell lysates compared with that in the p50-overexpressing cell lysates (Fig. 6E, comparing lanes 5 and 11), suggesting that hTERT enhances hTERT:p50:p50 complex binding to the PLEKHA7 promoter.

Next, we performed a supershift assay to evaluate hTERT and p50 binding to the PLEKHA7 promoter. Notably, the antibody against p50 and hTERT did not supershift the complexes because the p50 and hTERT binding site oligonucleotides were mutated (Fig. 6F, lanes 3 and 5), but the anti-hTERT and anti-p50 antibodies supershifted the complexes in the presence of their binding site oligonucleotides (Fig. 6F, lanes 2 and 4). As expected, both the anti-hTERT and anti-p50 antibodies showed higher-order supershift complexes, verifying their validity as complexes (Fig. 6F, lane 1). To further validate whether p50 and hTERT can bind the “GGGGAGCGCC” sequence and “CTGGG” sequence in the PLEKHA7 promoter, the “GGGGAGCGCC” sequence, “CTGGG” sequence or both motifs in the PLEKHA7 promoter were mutated by the CRISPR–Cas9 method in MKN74 cells (Fig. S7I–K). We detected significantly inhibited levels of hTERT binding or p50 binding in the “ GGGGAGCGCC” sequence - or “ CTGGG” sequence -knockout cells, and the effect was the greatest in the dual-motif-knockout cells compared with that in the control cells (Fig. 6G, H). Thus, our data demonstrate that hTERT and p50 directly bind the PLEKHA7 promoter and that hTERT exhibits tighter binding and can inhibit PLEKHA7 transcription better than p50.

### hTERT plays a major role in suppressing PLEKHA7 expression and PLEKHA7 deficiency-induced migration/invasion

**Fig. 7:**
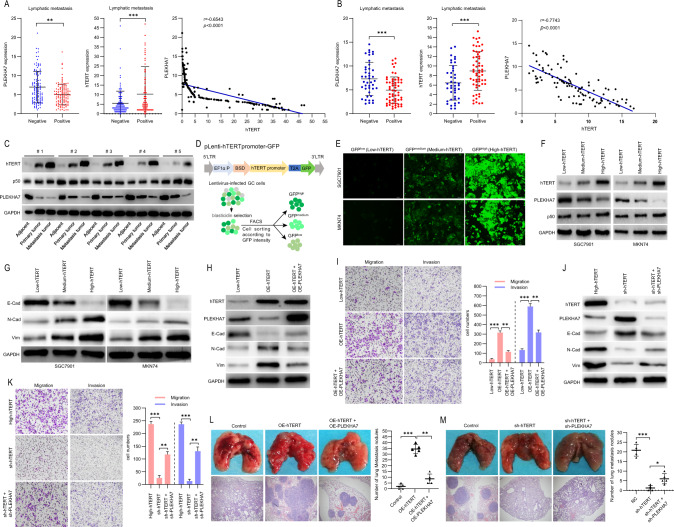
hTERT plays a major role in the hTERT/p50 repressor complex. **A** The expression of PLEKHA7 (left) and hTERT (middle) and their correlation (right) in The Cancer Genome Atlas (TCGA)-STAD cohorts. **B** The expression of PLEKHA7 (left) and hTERT (middle) and their correlation (right) in the gastric cancer (GC) tissues we collected. **C** Western blot analysis was performed to detect hTERT, p50 and PLEKHA7 expression in 5 groups of paired adjacent, primary and metastatic GC tissues. **D** Schematic showing the screening of the lentivirus (Lv)-hTERT-promoter-GFP reporter system. GFP expression was controlled by the hTERT promoter. GC cells infected with the lentiviruses were subjected to 2 weeks of blasticidin selection. GFP^low^, GFP^medium^ and GFP^high^ GC cells were then selected by fluorescence-activated cell sorting (FACS). **E** GFP images of hTERT^low^, hTERT^middle^ and hTERT^high^ MKN74 cells and SGC7901 cells. **F** The expression of hTERT, PLEKHA7 and p50 in hTERT^low^, hTERT^middle^ and hTERT^high^ MKN74 and SGC7901 cells. **G** Western blot experiments showing the epithelial-to-mesenchymal transition (EMT)-associated protein levels in hTERT^low^, hTERT^middle^ and hTERT^high^ MKN74 and SGC7901 cells. **H** The expression of PLEKHA7 and EMT-associated proteins in low-hTERT MKN74 cells after overexpressing hTERT (OE-hTERT) and PLEKHA7 (OE-PLEKHA7). **I** hTERT overexpression (OE-hTERT) in low-hTERT MKN74 cells enhanced MKN74 cell migration and invasion abilities versus those in low-hTERT MKN74 cells, and PLEKHA7 overexpression (OE-PLEKHA7) in OE-hTERT MKN74 cells partially decreased MKN74 cell migration and invasion abilities versus OE-hTERT MKN74 cells. **J** The expression of PLEKHA7 and EMT-associated proteins in high-hTERT MKN74 cells after knocking down hTERT (sh-hTERT) and PLEKHA7 (sh-PLEKHA7). **K** Knocking down hTERT (sh-hTERT) in high-hTERT MKN74 cells decreased MKN74 cell migration and invasion abilities versus high-hTERT MKN74 cells, and knocking down PLEKHA7 (sh-PLEKHA7) in high-hTERT MKN74 cells partially enhanced MKN74 cell migration and invasion abilities versus knockdown-hTERT (sh-hTERT) MKN74 cells. **L**, **M** A mouse metastasis model was established by intravenous injection of hTERT-overexpressing (OE-hTERT) cells and PLEKHA7-overexpressing (OE-PLEKHA7) cells or hTERT-knockdown (sh-hTERT) cells and PLEKHA7-knockdown (sh-PLEKHA7) cells. Left top: the number of metastatic nodules in the lung. Left bottom: representative images of haematoxylin and eosin (H&E)-stained lung. Right: bar graphs of tumour nodules.

As hTERT exhibits tighter PLEKHA7 promoter binding than p50, we investigated the effect of hTERT in regulating PLEKHA7 expression. The western blot analysis showed that PLEKHA7 expression decreased with increased hTERT expression compared with that in the vector control (Fig. 6I). Through analyses of The Cancer Genome Atlas (TCGA) STAD dataset and GC patient samples we collected, we found that the PLEKHA7 mRNA levels were significantly negatively correlated with the hTERT mRNA levels in metastatic GC tissues (Fig. 7A, B). The protein level analysis of the tissues (adjacent tissue, primary tumour tissue, and metastatic tumour tissue from the same GC patients) showed that the p50 protein level slightly fluctuated, while the hTERT protein level gradually increased, and the PLEKHA7 protein level gradually decreased from adjacent tissue to metastatic tissue (Fig. 7C). These results further suggest that hTERT plays a key role in the hTERT:p50:p50 complex inhibiting PLEKHA7 expression. To validate the above supposition, we constructed an hTERT-promoter-GFP lentiviral system in which GFP expression was driven by the hTERT “core promoter” as previously reported [37] to isolate different subtypes of GC cells with differing hTERT expression levels (hTERT^low^, hTERT^medium^ and hTERT^high^). Then, MKN74 and SGC7901 cells were infected with a lentivirus, and cells stably expressing GFP were selected with blasticidin (Fig. 7D). We performed fluorescence-activated cell sorting (FACS) to select approximately 10% of the most intensely GFP-positive (GFP^high^), approximately 10% of the intermediately intense GFP-positive (GFP^medium^) and approximately 10% of the least intense GFP-positive (GFP^low^) MKN74 and SGC7901 cells (Fig. 7E). Compared with the GFP^low^ and GFP^medium^ cell sets, the GFP^high^ cells exhibited higher hTERT protein levels and more significant differences in EMT candidate proteins (Fig. 7E–G), indicating that the hTERT promoter-driven reporter system faithfully reflected endogenous hTERT expression. We also observed that decreased PLEKHA7 was associated with increased hTERT (Fig. 7F).

Several studies have shown that forced hTERT expression upregulates the invasion/migration ability of GC cells [2, 4, 16]. Considering these previous studies and our study results (Figs. 2 and 4), we speculated that hTERT promoted GC cell invasion/metastasis, at least partially, through the hTERT/p50-PLEKHA7 axis. To validate this hypothesis, we overexpressed hTERT and PLEKHA7 or only hTERT in hTERT^low^ MKN74 cells to investigate the influence of their expression on GC cell migration and invasion abilities (Fig. 7H). We performed Transwell assays and observed that the migration and invasion abilities of the hTERT^low^ MKN74 cells increased after hTERT overexpression and that this ability was partially inhibited after PLEKHA7 was overexpressed (Fig. 7I). In contrast, we found that decreased hTERT expression reduced the migration and invasion abilities of hTERT^high^ MKN74 cells and that these reduced migration and invasion capacities were partially rescued after PLEKHA7 expression was knocked down in hTERT^high^ MKN74 cells (Fig. 7J, K). These results reveal that the increase in hTERT^high^ MKN74 cell migration and invasion abilities partially depended on PLEKHA7 expression. Then, an animal metastatic model experiment was performed to investigate the effects of hTERT and PLEKHA7 expression on GC tumour migration. MKN74 cells expressing differing levels of hTERT and PLEKHA7 established through gene interference or overexpression were injected into the tail vein of nude mice. The results showed that the number of lung metastasis nodules in the hTERT overexpression group (OE-hTERT) was significantly higher than that in the control group (*P* < 0.001) (Fig. 7L), which is consistent with previous reports [16]. Moreover, PLEKHA7 overexpression significantly attenuated hTERT-induced metastasis in vivo (Fig. 7L). In contrast, the PLEKHA7 knockdown partially abolished the sh-hTERT-attenuated metastasis (Fig. 7M). These results confirm that hTERT is critical for the hTERT:p50:p50 complex inhibition of PLEKHA7 expression and PLEKHA7 deficiency-induced migration/invasion.

### The hTERT-PLEKHA7 interaction is associated with metastatic progression in GC patients

**Fig. 8:**
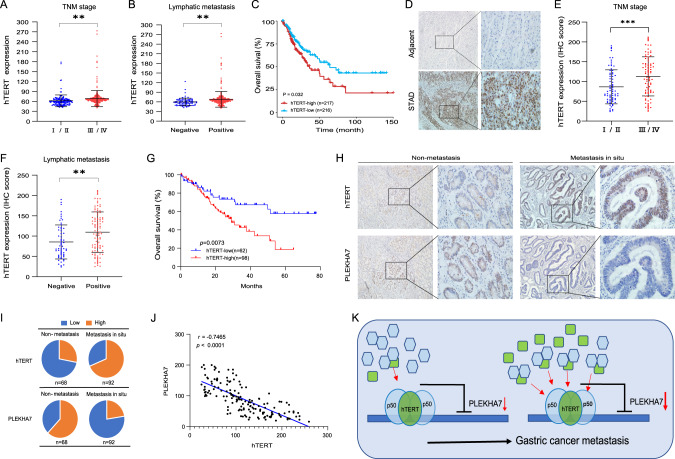
The hTERT/PLEKHA7 axis is associated with malignant progression in gastric patients. **A** hTERT mRNA levels were increased in stage III/IV gastric tumours compared with those in stage I/II gastric tumours in The Cancer Genome Atlas (TCGA)-STAD cohorts. **B** hTERT mRNA levels were increased in lymphatic metastasis gastric tumours compared with those in nonlymphatic metastatic tumours in TCGA-STAD cohorts. **C** Survival analysis showing that overall, patients with high hTERT expression exhibited lower overall survival than those with low hTERT expression. **D** Representative immunohistochemistry (IHC) image showing stained hTERT protein in gastric tumours and paired adjacent tissues. **E** hTERT protein levels were significantly higher in stage III/IV gastric tumours than in stage I/II gastric tumours. **F** hTERT protein levels in lymphatic metastatic gastric tumours were significantly higher than those in nonlymphatic metastatic tumours. **G** Survival analysis showing that overall, patients with high hTERT expression exhibited lower overall survival than those with low hTERT expression. **H** Representative immunohistochemistry (IHC) image showing stained hTERT protein and PLEKHA7 protein in corresponding gastric tumour tissue. **I** Percentages of low and high expression of hTERT (upper) and PLEKHA7 (bottom) in nonmetastatis GC tissues and metastasis GC tissues. **J** Spearman’s correlation of the hTERT and PLEKHA7 protein levels. **K** Schematic diagram summarizing the findings of the present study.

We further investigated the role of hTERT in GC. Specifically, we assessed the hTERT expression levels using a publicly available GC dataset (GSE84437) and the IHC results we obtained from GC cohort 1 tissues. We observed that GC patients with stages III/IV and lymphatic metastasis showed higher hTERT RNA and protein levels than those with stages I/II and nonlymphatic metastasis (Fig. 8A, B, E, F). We also observed aberrantly high levels of hTERT protein in the tumours compared with those in the adjacent tissue (Fig. 8D). The survival analysis revealed that patients with high hTERT RNA and protein levels had shorter overall survival times than those with low hTERT expression levels (Fig. 8C, G).

To determine whether hTERT/PLEKHA7 axis-mediated metastasis is clinically relevant, 160 nonmetastatic and metastatic tissue samples obtained from GC patients were subjected to IHC staining. We found that metastatic GC tissues with high hTERT staining often displayed low PLEKHA7 staining compared to the nonmetastatic GC tissues (Fig. 8H). The IHC statistical results showed more number of high hTERT staining in metastatic GC tissues than in nonmetastatic GC tissues and less number of low PLEKHA7 staining in metastatic GC tissues than in nonmetastatic GC tissues (Fig. 8I). In addition, the expression of hTERT was negatively related to PLEKHA7 expression (Fig. 8J). Altogether, these data suggest that the hTERT–PLEKHA7 interaction is associated with metastatic progression in GC patients (Fig. 8K).

## Discussion

In this study, the expression of PLEKHA7 gradually decreased with increased GC malignancy, inspiring us to study the molecular mechanism of this action. To confirm that reduced PLEKHA7 expression contributes to gastric tumour progression, we performed a range of biochemical and pathological statistical analyses, and the results showed that patients with lower PLEKHA7 expression exhibited lower overall survival. In addition, PLEKHA7 deficiency was significantly correlated with distant metastasis. Increased GC cell migration and invasion, specifically lung metastatic ability in animal models, were also confirmed by knocking down PLEKHA7 expression. PLEKHA7 was originally identified as an adaptor protein connecting AJs to the end of microtubules and a ZA stabilizer [22]. PLEKHA7 was subsequently found to be an interactor of p120-catenin [25] and paracingulin expression [20, 21]. It has been reported that PLEKHA7 interacts with microtubules through the Nezha/CAMSAP3 protein, stabilizes AJs and promotes epithelial barrier function [24, 26]. These findings have increased interest in the possible association between PLEKHA7 expression and the pathogenesis of hypertension [28, 34] and glaucoma [38] because several cytoskeletal PLEKHA7 partners play important roles in both pathological diseases. Moreover, PLEKHA7 has been shown to be involved in regulating miRNA levels, which are critical for the suppression of tumorigenic signalling [30] and Rho GTPase activity [31]. The results of this and previous studies have shown the loss of PLEKHA7 expression in tumour tissues [26, 31] and elucidated the roles that PLEKHA7 plays in regulating tumourigenesis and metastatic miRNA levels [24, 30, 32], implicating PLEKHA7 in critical regulatory tumour invasion and metastasis mechanisms. Therefore, investigations of the molecular mechanism of PLEKHA7 loss in tumours may be important for gaining a comprehensive understanding of tumour invasion and metastasis.

Two different transcription factor prediction websites were accessed, and DNA pull-down experiments were performed to identify transcription factors that might regulate PLEKHA7 expression. The intersection of the transcription factors obtained through these two inquiries was subjected to further analysis. Intriguingly, knocking down p50 expression significantly increased PLEKHA7 expression, indicating that p50 might regulate PLEKHA7 expression. Furthermore, PLEKHA7 promoter activity was increased after p50 was knocked down and was significantly reduced when the putative p50-binding site in the PLEKHA7 promoter was mutated. Then, p50 binding to the PLEKHA7 promoter was confirmed by a ChIP analysis in GC cells. These data demonstrate that PLEKHA7 is directly transcriptionally regulated by p50. Notably, p50 belongs to the NF-κB family and generally refers to a p65/p50 heterodimer, which is among the most ubiquitous dimers and the major Rel complex in the NF-κB family [39, 40]. This complex is constitutively active as a transcriptional activator in different types of human tumours and facilitates the expression of genes that increase cell proliferation and antiapoptotic functions [41]. In addition, p50 has no transactivation domain to activate transcription and, thus, is considered a transcriptional repressor [36, 42]. Interestingly, PLEKHA7 expression remained unchanged when p65 expression was knocked down, indicating that PLEKHA7 regulation was not mediated through the canonical p65/p50 pathway. When we forced p50 expression, PLEKHA7 expression was reduced by approximately 10% compared with that in the control. Altogether, these results indicate that factors other than p50 coregulate PLEKHA7 expression.

We searched for proteins that might interact with p50 by performing co-IP experiments. Several proteins were obtained via co-IP and subjected to further analysis. Several putative hTERT-binding sites in the PLEKHA7 “core promoter” were found. Subsequently, dual luciferase reporter assays confirmed that the region from 233 bp to 237 bp upstream of the PLEKHA7 “core promoter” is the hTERT-binding site. Interestingly, the position next to the hTERT-binding site is the p50-binding site (from 251 bp to 261 bp upstream of the PLEKHA7 “core promoter”) in the PLEKHA7 “core promoter”. Moreover, hTERT overexpression significantly decreased PLEKHA7 expression, which was rescued after hTERT expression was knocked down in GC cells. The physical interaction between hTERT and p50 was also revealed in our study. hTERT is not an independently active transcription factor and does not regulate gene expression unless it cooperates with other transcription factors. hTERT has been previously shown to recruit the p65/p50 complex to regulate NF-κB-dependent gene expression [12], bind BRG1 to activate Wnt-dependent genes [43], and interact with c-myc to enhance heparinase expression [44]. Our data reveal that the hTERT/p50 complex synergistically suppressed PLEKHA7 expression by inhibiting PLEKHA7 transcription. hTERT overexpression led to enhanced hTERT interaction with p50, while hTERT inhibition by sh-hTERT reduced the formation of this complex. Furthermore, hTERT recruited p50 to form a transcriptional repressor complex and directly occupied a binding site on the PLAKH7 promoter, indicating that both hTERT and p50 are essential for the transcriptional suppression of PLEKHA7 expression in GC cells.

We found no difference in the abundance of p50 during GC progression, but hTERT expression gradually increased, especially in metastatic GC cells. Moreover, after p50 was overexpressed, PLEKHA7 expression decreased only by 10%. The low hTERT expression in GC cells likely contributes to the repressed formation of the hTERT/p50 suppressive complex, which results in a lack of PLEKHA7 inhibition. In contrast, hTERT overexpression significantly increased hTERT/p50 complex binding to the PLEKHA7 promoter and decreased PLEKHA7 expression. The functional rescue experiments further demonstrated that hTERT dominated the inhibitory effect of the p50/hTERT complex on PLEKHA7 expression in GC cells.

In conclusion, decreased PLEKHA7 expression was associated with a poor prognosis and distant tumour metastasis of GC cells. Moreover, the gradual decrease in PLEKHA7 expression was regulated by the hTERT/p50 complex, which is dominated by hTERT action, during gastric tumour metastasis. Altogether, our findings not only provide novel insight into the relationship between PELKHA7 and GC metastasis but also reveal that the hTERT/p50-PLEKHA7 axis may be a therapeutic target in GC.

## Materials and methods

### Cell lines and patient samples

Human GC cell lines (AGS, MKN74, MKN28, SGC7901, BGC823, MGC803 and MKN45 cell lines) and the GES-1 immortalized gastric epithelial cell line were purchased from and authenticated by the Type Culture Collection of the Chinese Academy of Sciences (Shanghai, China). The cells were cultured in DMEM (Gibco, USA) supplemented with 10% foetal bovine serum (FBS, Gibco, USA), 100 U/mL penicillin and 100 mg/mL streptomycin (Gibco, USA) in an incubator at 37 °C and 5% CO_2_.

Fresh GC tissues and paired adjacent noncancer tissues were obtained during gastrotomy surgeries performed at the Department of General Surgery, Xinqiao Hospital (Chong Qing, China). All samples were stored in liquid nitrogen and used after obtaining informed consent from each patient. The experimental protocol for the collection and use of the clinical tissues was approved by the Institutional Review Board of Xinqiao Hospital.

### Plasmids and cloning

Lentiviral constructs expressing shRNAs directed against PLEKHA7, hTERT, p50, p65, or POU2F2 and a negative control were obtained from Thermo Fisher Scientific. The gene knockdown efficiency was confirmed by RT–qPCR and a western blot analysis. PLEKHA7 (NM_175058), hTERT (NM_198253), p50 (NM_003998), p65 (NM_021975), and POU2F2 (NM_001207025) expression plasmids were generated by cloning the full-length open reading frame (ORF) into a pCMV6-Entry vector (GenScript), and these genes were used as a template to generate genes with different tags (pCMV6-Myc-hTERT, pCMV6-Flag-p50, and pCMV6-HA-p50). For the CRISPR/Cas9 experiments, single guide RNAs (sgRNAs) were cloned into a lentiCRISPR v2-puro plasmid (Addgene #98290). The p50 S342A mutant was constructed by two-step site-directed mutagenesis using pCMV6-p50 as a template according to the protocol described in the study by C. L. Wilson [36].

### RNA extraction and real-time PCR

The total RNA was isolated from tissues or cultured cells using a total RNA extraction kit (TaKaRa). cDNA was synthesized using random primers (TaKaRa). qPCR was performed using a QuantiTect SYBR Green PCR Kit (Qiagen).

### Chromatin immunoprecipitation

ChIP assays were performed using ChIP-IT^®^ Express chromatin immunoprecipitation kits (Active Motif Biotechnology). Each assay was performed using 30 µg of chromatin fragment and 6 µg of antibody. The enriched DNA in the PLEKHA7 promoter fragment was detected by qPCR using specific primers.

### DNA pull-down assays

DNA pull-down assays were performed as previously described [45]. Briefly, the 5′-biotinylated PLEKHA7 “core promoter (−2000 bp, 200 bp)” was amplified by PCR. The forwards primer, 5′-TACTTTTCTATTAACTGTGGGTTCATACCCT-3′, and reverse primer, 5′-GAGGTGCACCTGTTAGCGCCG-3′, were used. The 5′-biotinylated PLEKHA7 promoter DNA was immobilized on streptavidin beads using a Dynabeads® kilobase BINDERTM kit (Invitrogen). The cells were lysed, and the nuclear fractions were isolated. Then, the nuclear fraction proteins and 5′-biotinylated DNA–bead mixture were incubated overnight at 4 °C on a rotator. The protein–DNA beads were enriched and washed three times with cold PBS. Then, the proteins were eluted and subjected to polyacrylamide gel electrophoresis (PAGE) followed by a western blot analysis or mass spectrometry (MS) analysis. Proteins eluted from beads without the 5′-biotinylated DNA probe were used as controls.

### Protein extraction and western blot analysis

Cells or tissues were lysed with 1×SDS loading buffer and sonicated. The protein quantitation was performed with an RC DC protein assay kit (Bio-Rad). In total, 50 µg of protein per well were separated by 10% SDS–PAGE and transferred onto a PVDF membrane (Millipore, USA), which was blocked with 5% nonfat dry milk (Cell Signaling Technology, USA) before an overnight incubation with specific primary antibodies against hTERT (Abcam 1:1000 dilution), p50 (Abcam 1:2000 dilution), p65 (Cell Signaling 1:1000 dilution), POU2F2 (Cell Signaling 1:1000 dilution), E-cadherin (Abcam 1:2000 dilution), N-cadherin (Cell Signaling 1:1000 dilution), Vimentin (Abcam 1:2000 dilution), Myc (Abcam 1:2000 dilution), Flag (Proteintech 1:10000 dilution), HA (Proteintech 1:5000 dilution), p120 (Cell Signaling 1:1000 dilution), p-p120 (Y228) (Cell Signaling 1:2000 dilution), PLEKHA7 (Invitrogen 1:700 dilution), HistoneH3 (Abcam 1:2000 dilution), and GAPDH (Proteintech 1:10000 dilution) at 4 °C, followed by incubation with secondary antibodies (goat anti-rabbit IgG-HRP and goat anti-mouse IgG-HRP) (Proteintech 1:10000 dilution). The blots were visualized using enhanced chemiluminescence (Thermo Fisher Scientific).

### Immunohistochemistry

Paraffin-embedded tissue sections were used for IHC staining. The sections were deparaffinized in a 60 °C oven for 1 h, dewaxed three times in xylene for 5 min, and sequentially incubated with 95%, 80%, 70% and 50% graded ethanol for rehydration. Antigen retrieval was performed using EDTA-mediated high-temperature and high-pressure methods, and endogenous peroxidase activity was blocked by 3% H2O2 for 15 min. The sections were blocked with 10% goat serum and incubated overnight with specific primary antibodies against hTERT (Abcam 1:100 dilution), p50 (Cell Signaling 1:200 dilution), and PLEKHA7 (Invitrogen 1:100 dilution) at 4 °C. The sections were washed with TBST for 15 min (for 5 min per wash) at room temperature and then incubated with biotinylated goat anti-rabbit (sp-9000, ZsGB-Bio) for 20 min, followed by staining with a ZsGB-Bio DAB detection kit and counterstaining with haematoxylin before dehydration and mounting.

### Nuclear protein extraction and electromobility shift assays

Nuclear proteins were extracted using an NE-PER™ nuclear and cytoplasmic extraction reagent kit (Pierce). Briefly, the cells were lysed with Dignam CER I buffer, and then, Dignam CER II buffer was added to the lysate, which was incubated on ice for 1 min. After centrifugation, the supernatant (the cytoplasmic extract) was poured into a clean prechilled tube, resuspended in Dignam nuclear extraction reagent (NER) buffer and incubated on ice for 40 min. The nuclear extracts were collected after centrifugation and used to perform EMSAs. Each EMSA was performed with a LightShift chemiluminescent EMSA kit (Pierce). Briefly, for the PLEKHA7 EMSA, 10 µg of nuclear extract were incubated with biotin-labelled PLEKHA7 oligonucleotide (Invitrogen) at 4 °C for 30 min. For the supershift assays, 3 µg of anti-hTERT or anti-p50 antibody were added to the nuclear extracts, which were then incubated on ice for 30 min before the addition of biotin-labelled probes. A 6% nondenaturing polyacrylamide gel was used to resolve the supershift reaction mixtures.

### Migration and invasion assays

Transwell assays were performed to assess the migration and invasion abilities of the cells. For the migration assay, the cells were harvested after culturing for 24 h in serum-free DMEM, and these harvested cells were seeded into the upper Transwell chamber in DMEM containing 0.1% FBS (300 µl, 2 × 10^5^ cells). Then, 400 μL of DMEM containing 20% FBS were added to the lower chamber and served as a chemoattractant. The migrating cells were fixed with 4% paraformaldehyde, stained and photographed after incubation for 48 h. For the invasion assay, the Transwell chambers were coated with Matrigel (Millipore, Billerica, Massachusetts, USA), and a protocol similar to that used for the migration assay was followed. Finally, the migrated or invaded cells in five random fields on the evaluated filters were counted.

### Mouse metastasis model

A 200 µL volume of cells (2 × 10^6^) was injected into the tail vein of 5-week-old male BALB/C nude mice (Shanghai Laboratory Animal Center, Shanghai, China). Forty days after inoculation, the lungs were collected, and the metastatic nodules were counted, stained with H&E and photographed. The experimental protocol for the collection and use of the BALB/C nude mice was approved by the Institutional Review Board of Xinqiao Hospital.

### Coimmunoprecipitation assay

Co-IP was performed using a universal magnetic co-IP kit (Active Motif Biotechnology) following the manufacturer’s instructions. Briefly, cells on 10 cm plates were lysed with 100 μL of complete whole-cell lysis buffer, incubated on ice for 30 min, and then centrifuged at 16,000 × *g* for 10 min at 4 °C. The cell lysates (300 µg of whole-cell extract per reaction) were incubated with 5 µg of specific primary antibodies overnight on a rotator at 4 °C. On the following day, 25 µl of protein A/G magnetic beads were added to each tube, and the sample was incubated for 1 h on a rotator at room temperature. The protein beads were washed with complete co-IP/wash buffer four times, eluted using 2×SDS loading buffer and analysed by immunoblotting.

### Dual-luciferase reporter assay

To generate the PLEKHA7 promoter (from −2000 to +200) firefly luciferase reporter, promoters of wt and mutant PLEKHA7 were synthesized by Sangon Biotech and cloned into a pGL3-reporter vector. Five replicate MKN74 cells were seeded in 48-well plates at 1.5 × 10^5^ cells per well. On the following day, 50 ng of reporter plasmids and 10 ng of Renilla luciferase control plasmids (pRL-TK) were cotransfected into MKN74 cells using Lipofectamine 3000 (Invitrogen). After 48 h, the cells were collected, and luciferase activity was measured using a Dual-Luciferase Reporter assay system (Promega).

### Nuclear run-on assay

A nuclear run-on assay was performed using previously described methods [46] to determine whether PLEKHA7 expression induced by p50 or hTERT was caused by transcriptional regulation. Nuclei were extracted from MKN74 cells (5◊10^7^) after different treatments in NP-40 lysis buffer. The nuclear extracts were resuspended in 500 µL of nuclear freezing buffer and then mixed thoroughly with 200 µL of 5◊run-on buffer. Then, 8 µL of biotin-16-UTP (11388908910, Sigma–Aldrich) were added to the nuclei, and the sample was incubated for 20 min at 37 °C. The nuclear run-on reaction was quenched with 1.5 µL of 1 M CaCl2. RNA was purified with an RNeasy mini kit (74104, QIAGEN) and divided into small aliquots. The RNA samples were mixed with Dynabeads M-280 with streptavidin (11205D, Thermo Fisher Scientific) and incubated at 37 °C for 2 h on a rotator. Then, the RNA samples were washed with 2× saline sodium citrate (SSC) 3 times, and the beads were isolated and resuspended in 20 µL of nuclease-free water. RT–qPCR was performed, and the PCR products were separated on a 1.5% agarose gel.

### Statistical analysis

The data were analysed with SPSS 20.0 software (SPSS, USA) or GraphPad Prism 8.0 software (GraphPad Prism, USA) and are presented as the means ± SEMs or the means ± SDs. PLEKHA7 IHC expression in paired adjacent or normal mucosal tissue and cancer tissue was analysed by paired *t* tests. The correlations between PLEKHA7 IHC expression and the categorical clinicopathological variables were evaluated by a Pearson’s chi-square test. The survival analyses were performed using the Kaplan–Meier method. The data shown in bar graphs are presented as the means ± SEMs, and *p* values <0.05 were considered significant (^*^p < 0.05, ^**^p < 0.01, ^***^p < 0.001).

## Supplementary information

Figure S1-S7 legends

Figure S1

Figure S2

Figure S3

Figure S4

Figure S5

Figure S6

Figure S7

## Data Availability

The datasets supporting the conclusions of this article are included within the article and its additional files. The RNA-seq data can be accessed by GEO series accession number GSE84437.
