# Supplementary material for: The hTERT-p50 homodimer inhibits PLEKHA7 expression to promote gastric cancer invasion and metastasis
Source: Oncogene. 2023 Feb 23;42(14):1144–56. doi: 10.1038/s41388-023-02630-9 (PMC10063444; doi:10.1038/s41388-023-02630-9)
Supplement: Supplementary file 1 — Figure S1-S7 legends [file 41388_2023_2630_MOESM1_ESM.docx]

**Supplementary Figure 1. PLEKHA7 deficiency in GC gradually decreases with GC progression**

(A) Screening of PLEKHA7 expression levels using immunohistochemistry (IHC) staining in 6 types of tumours identified significantly low PLEKHA7 expression in stomach tumour tissue compared with that in adjacent tumour tissue.

(B) Negative and weak PLEKHA7 IHC-stained tissues were classified as having low PLEKHA7 expression, and moderate to strong PLEKHA7 IHC staining was classified as having high PLEKHA7 expression.

(C) IHC staining of PLEKHA7 in different stages of GC tissue.

(D) IHC staining of PLEKHA7 in nonmetastatic gastric tumour tissue and metastatic gastric tumour tissue.

**Supplementary Figure 2.** **The phenotype of AGS and MGC803 cells when PLEKHA7 was knockout by CRISPR/Cas9**

(A) The PLEKHA7 gene of the indicated cell lines were edited by different sgRNA-guided CRISPR/Cas9. The WT indicated the original PLEKHA7 gene sequence and the Edited indicated the modified PLEKHA7 gene sequence after application of CRISPR/Cas9.

(B) Western blot was used to detect the expression of PLEKHA7 after CRISPR/Cas9 applied in AGS and MGC803 cells.

(C) Transwell assay was applied to detect the migration and invasion ability of AGS and MGC803 cells after PLEKHA7 was knockout by CRISPR/Cas9.

**Supplementary Figure 3. Reduced PLEKHA7 expression in GC cells does not depend on mRNA or protein degradation**

(A, B) western blot analysis of PLEKHA7 in MKN74 and GES-1 cells treated with 10 µM cycloheximide (CHX) at the indicated time points (A); The PLEKHA7 band intensity was quantified by ImageJ after normalization to GAPDH and setting the t = 0 time point (B).

(C, D) western blot analysis of PLEKHA7 in MKN74 and GES-1 cells treated with 5 µM MG-132 at the indicated time points (C); The PLEKHA7 band intensity was quantified by ImageJ after normalization to GAPDH and setting the t = 0 time point (D).

(E) The decay of PLEKHA7 mRNA was monitored in MKN74 and GES-1 cells treated with cycloheximide.

(F) Determination of the PLEKHA7 transcription initiation rate was determined via a nuclear run-on assay of MKN74 GC cells and GES-1 cells.

**Supplementary Figure 4. Workflow of the identification of proteins binding DNA in the PLEKHA7 promoter region**

(A) PCR products of the 5’-biotinylated DNA of the PLEKHA7 promoter used as probes in the DAN pull-down assay.

(B) western blot analysis of GAPDH and histone H3 (a protein marker of the nuclear fraction) to validate the nuclear fraction and cytosolic fraction purity.

(C) Schematic showing the improved DNA pull-down assay.

(D) Improved DNA pull-down assay that was used to identify specific p50 and hTERT binding to the PLEKHA7 promoter in MKN74 GC cells.

**Supplementary Figure 5. PLEKHA7 expression is not regulated by p65, POU2F2 or p50 overexpression**

(A) Quantitation of the PLEKHA7 mRNA abundance after p50 overexpression.

(B) western blot analysis of PLEKHA7 expression after overexpressing p50.

(C) Determination of the PLEKHA7 transcription initiation rate by nuclear run-on assay in MKN74 cells after p50 overexpression for 72 h.

(D) Luciferase reporter assays were performed to analyse PLEKHA7 promoter transcriptional activity after p50 was overexpressed.

(E, F) Quantitation of the PLEKHA7 mRNA abundance after p65 (E) or POU2F2 (F) was overexpressed.

(G, H) western blot analysis of PLEKHA7 expression after p65 (G) or POU2F2 (H) was knocked down.

**Supplementary Figure 6. Expression of different tagged genes**

(A) western blot analysis of myc-hTERT expression after the overexpression of myc-hTERT.

(B) western blot analysis of Flag-p50 expression after the overexpression of Flag-p50.

(C) western blot analysis of HA-p50 expression after the overexpression of HA-p50.

(D) Schematic showing the mutation of p50 at S342.

(E) western blot analysis of Flag-p50S342A expression after the overexpression of Flag-p50S342A.

(F) Schematic showing the DNA region of p50 gene in the indicated MKN74 cell was knockout.

(G) western blot analysis of p50 expression after the p50 knockout.

**Supplementary Figure 7. hTERT- and p50-binding sites in the edited PELKHA7 promoter**

(A) Schematic showing PCR amplification of different regions in the PLEKHA7 promoter.

(B) The luciferase activity of wild-type (wt) and mutant PLEKHA7 promoters in MKN74 cells was detected by a luciferase reporter assay. The wt and mutant PLEKHA7 promoter reporter constructs used in this study (left) and the luciferase activity (right).

(C-H) Schematic showing p50- or hTERT-binding site mutations in the PLEKHA7 promoter. The p50-binding site mutation (C), the hTERT-binding site mutation (D), and both p50- and hTERT-binding site mutations (E); the p50-binding site was mutated into the p50 consensus sequence (F), the hTERT-binding site was mutated into the hTERT consensus sequence (G), and both p50- and hTERT-binding sites were mutated into the p50 and hTERT consensus sequences (H).

(I) Schematic showing that the hTERT-binding site in the PLEKHA7 promoter of the indicated MKN74 cell lines was knocked out.

(J) Schematic showing that the p50-binding site in the PLEKHA7 promoter of the indicated MKN74 cell lines was knocked out.

(K) Schematic showing that the p50- and hTERT-binding sites in the PLEKHA7 promoter of the indicated MKN74 cell lines was knocked out.
