# Supplementary figures and images for: The hTERT-p50 homodimer inhibits PLEKHA7 expression to promote gastric cancer invasion and metastasis
Source: Oncogene. 2023 Feb 23;42(14):1144–56. doi: 10.1038/s41388-023-02630-9 (PMC10063444; doi:10.1038/s41388-023-02630-9)

A

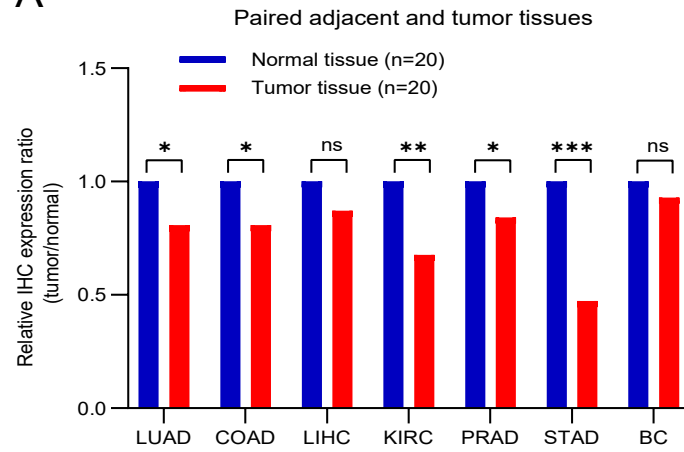

B

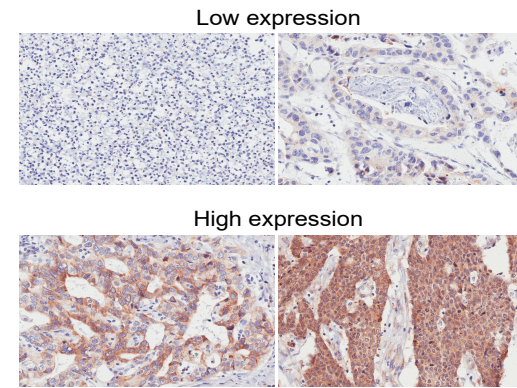

C

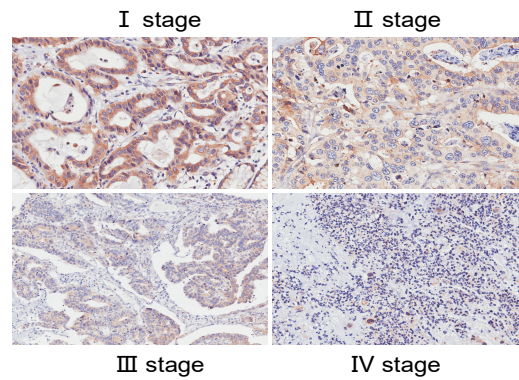

D

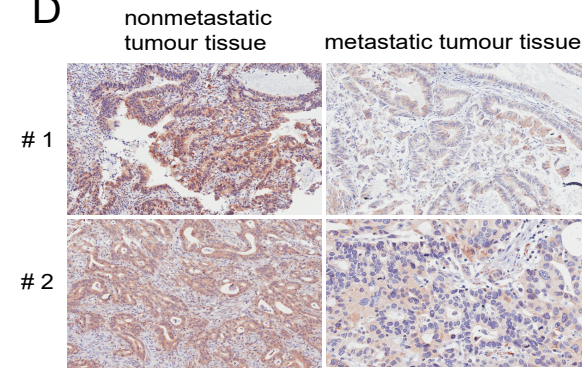

Supplement: Supplementary file 2 — Figure S1 [file 41388_2023_2630_MOESM2_ESM.pdf]

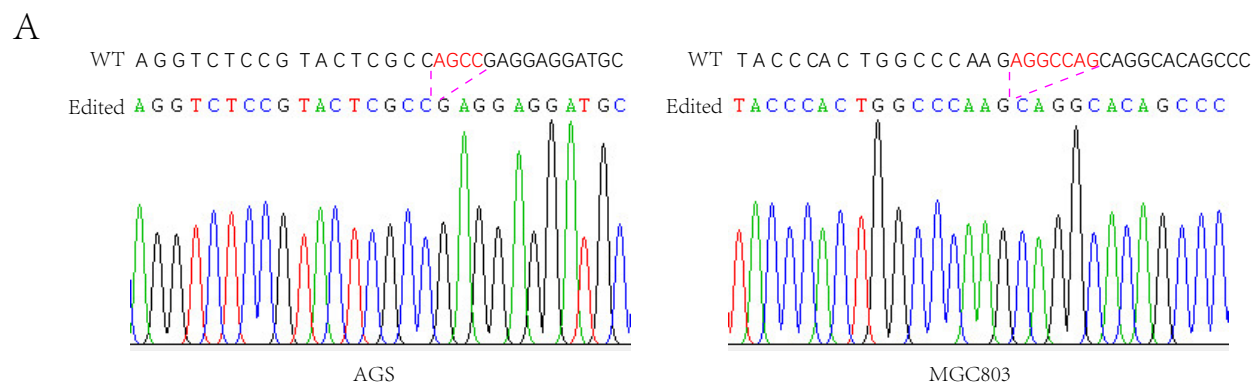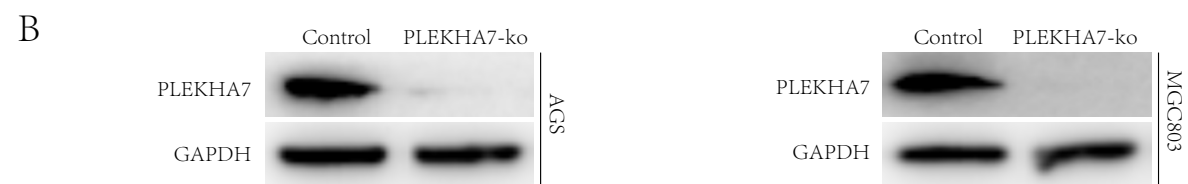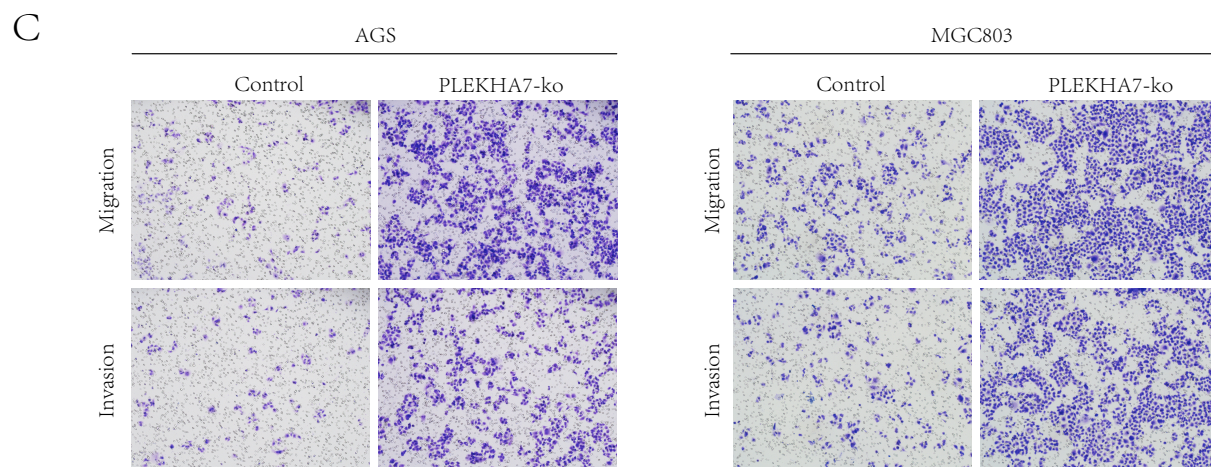

Supplement: Supplementary file 3 — Figure S2 [file 41388_2023_2630_MOESM3_ESM.pdf]

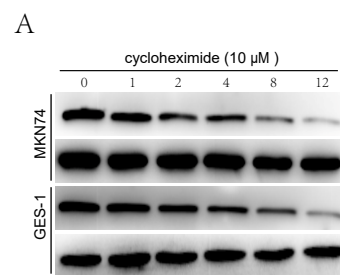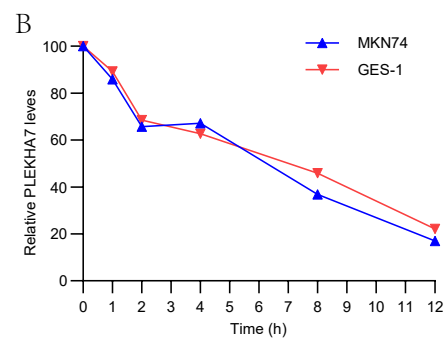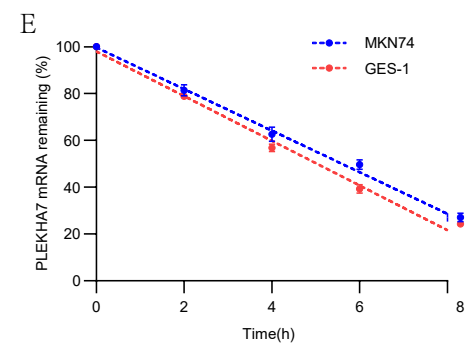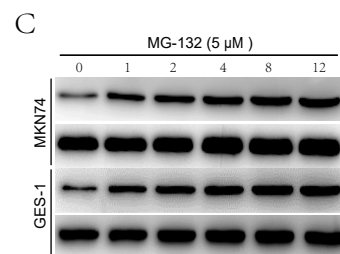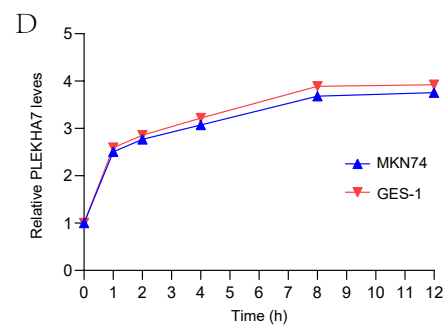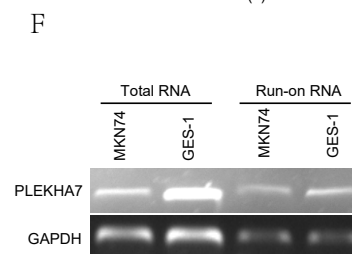

Supplement: Supplementary file 4 — Figure S3 [file 41388_2023_2630_MOESM4_ESM.pdf]

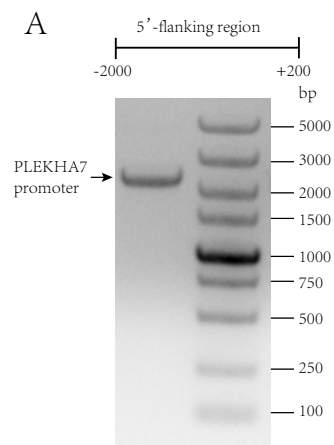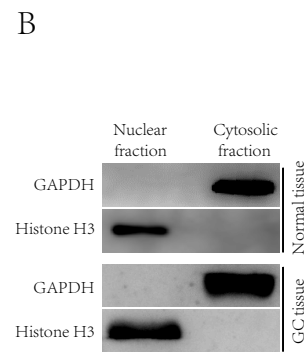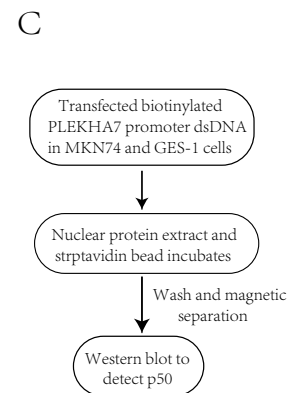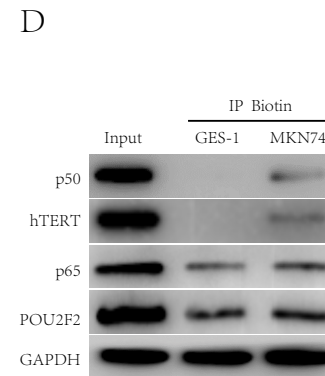

Supplement: Supplementary file 5 — Figure S4 [file 41388_2023_2630_MOESM5_ESM.pdf]

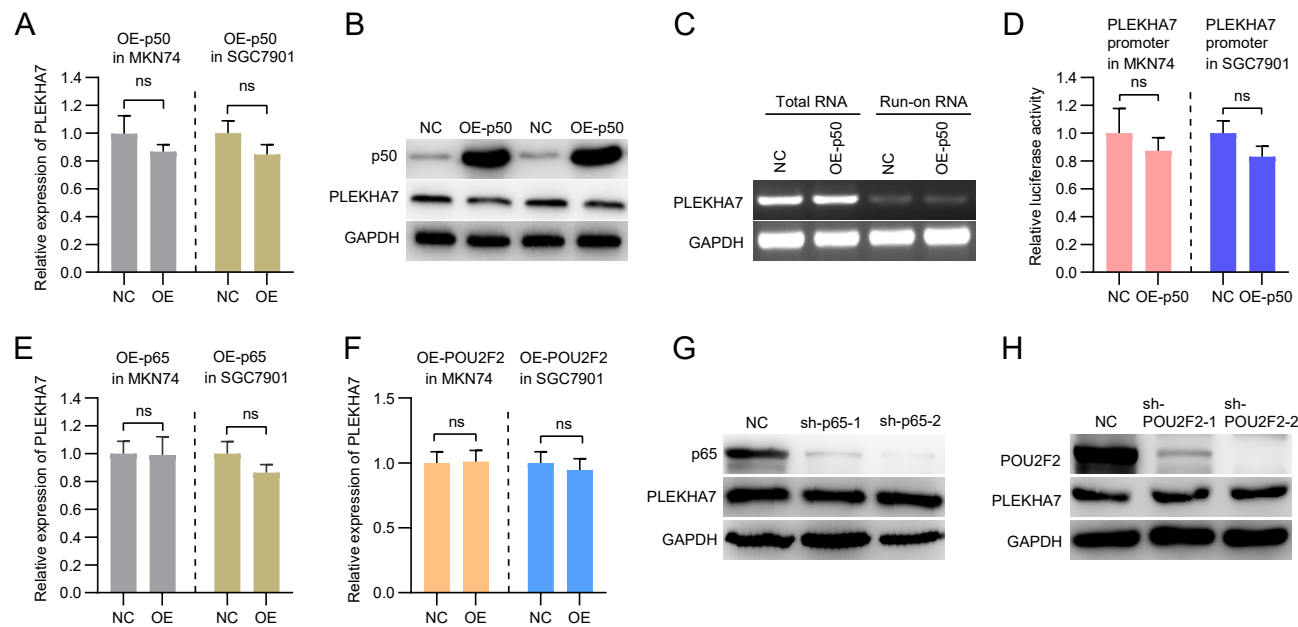

Supplement: Supplementary file 6 — Figure S5 [file 41388_2023_2630_MOESM6_ESM.pdf]

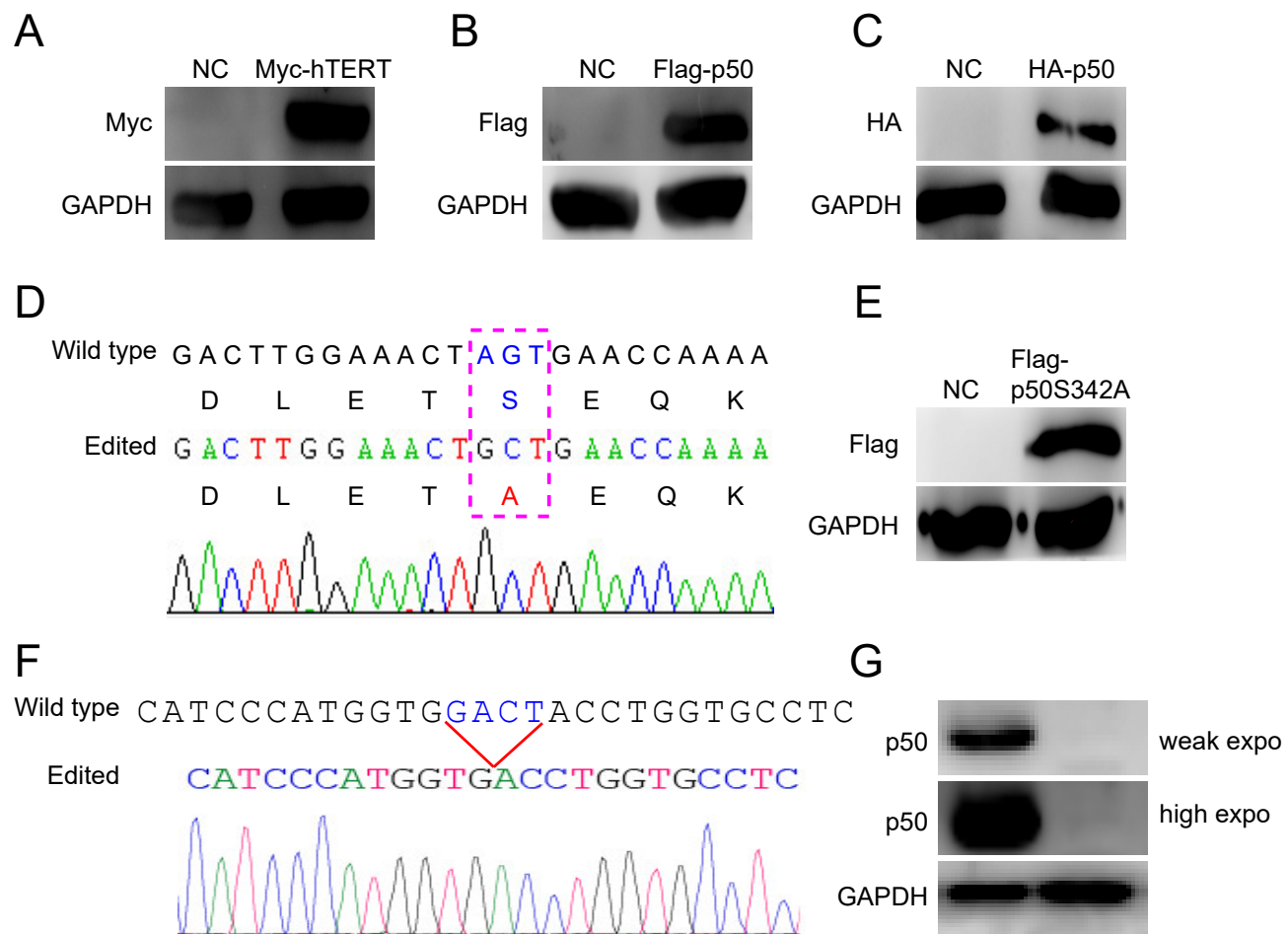

Supplement: Supplementary file 7 — Figure S6 [file 41388_2023_2630_MOESM7_ESM.pdf]

A

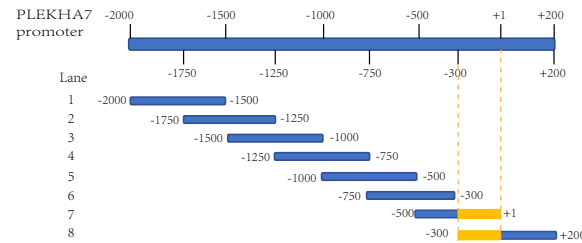

B

PLEKHA7 promoter reporter (-2000 to TSS)

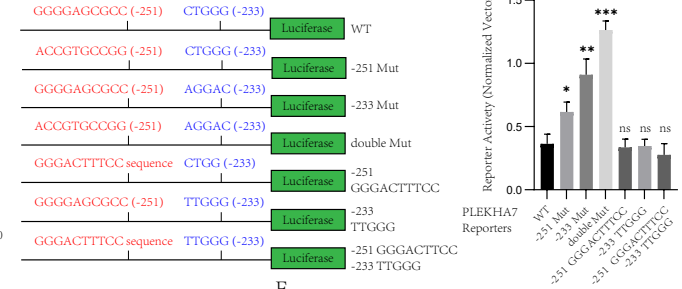

C

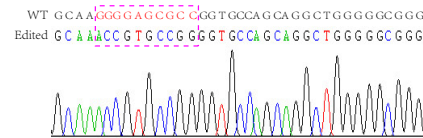

D

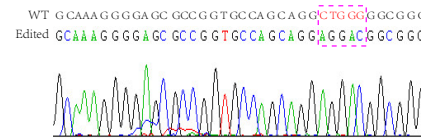

E

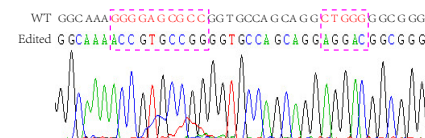

F

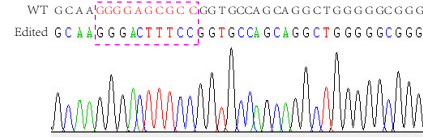

G

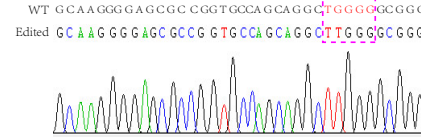

H

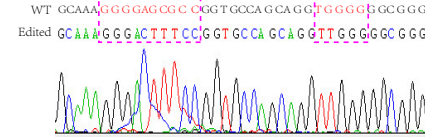

I

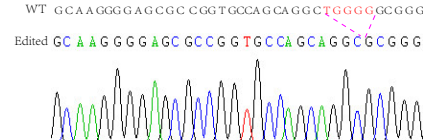

J

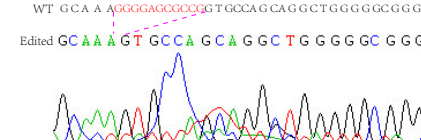

K

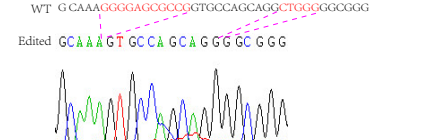

Supplement: Supplementary file 8 — Figure S7 [file 41388_2023_2630_MOESM8_ESM.pdf]
